# Correlation of cholesteatomas perimatrix thickness with patient's age

**DOI:** 10.1016/S1808-8694(15)31250-7

**Published:** 2015-10-20

**Authors:** Cristina Dornelles, Sady Selaimen da Costa, Luíse Meurer, Cláudia Schweiger

**Affiliations:** Master in Medical Sciences, Pediatrics, Ph.D. studies under course, Program of Post-Graduation in Medical Sciences: Pediatrics - UFRGS (Biologist, Centro de Otite Média do Brasil); Ph.D. in Surgery (Joint Professor); Ph.D. in Gastroenterology (Deputy Professor); Physician (Resident physician in Otorhinolaryngology). Hospital de Clínicas de Porto Alegre - Federal University of Rio Grande do Sul

**Keywords:** collagen, perimatrix

## Abstract

Cholesteatoma may occur either in children as in adults; in children, however, they have a more aggressive and extensive growth.

**Objective:**

To compare the thickness of the perimatrix, in μm, between adults (> 18 years old) and children cholesteatomas.

**Study design:**

transversal cohort.

**Material and Method:**

74 cholesteatomas (35 of children) obtained from othologic surgeries were included, fixed in formol 10%. It was made laminas with HE and Picrossisius, with were studied at the optic microscope. We obtained digital images of the laminas at the Image Pro-Plus and we used Spearman's coefficient for data analysis. Differences were considered statistically significant if P.

## INTRODUCTION

Cholesteatomas were defined by Schuknecht^1^ as the accumulation of exfoliated keratin in the middle ear or any pneumatized area of the temporal bone, rising from the keratinized squamous epithelium. It may affect both children and adults, but there is controversy about its clinical behavior in the different age ranges in which it is manifested.

According to Sheehy^2^, Tos^3^ and Edelstein^4^, pediatric cholesteatoma is less expansive, which leads to lower incidence of complications. Conversely, Galsscock^5^, Ruah^6^, Bujia^7^, Palva^8^ and Sudhoff^9^ report that acquired cholesteatoma in children should be presented in a more aggressive way and with more extensive growth. In the extreme of this disagreement, there is Smythe et al.^10^ that consider clinical differences between children and adults to be so massive that they think they should be distinct diseases.

Histologically, the cholesteatoma is comprised by perimatrix, matrix and cystic content^11^. According to these authors, the cholesteatoma perimatrix is subepithelial connective tissue that contains collagen fibers, elastic fibers, fibroblasts and inflammatory cells. They state that all cholesteatomas present perimatrix, but some are extremely thin, which can only be visualized by transmission electron microscopy and they confirmed that the reduction of thickness was resultant from reduction of collagen fibers and stated that this finding could indicate greater action of collagenase, enzymes that act on the lyses of these fibers.

Abramson^12^ demonstrated the presence of collagenase in cholesteatomas. Later, Thompsen^13^ demonstrated that this enzyme was produced by keratinocytes. Thus, the activity of collagenase could explain the power of bone erosion that cholesteatomas normally have. In 1997, Ferlito et al.^14^ suggested that bone erosion was caused by the production of this enzyme in squamous and fibrous epithelial tissues of cholesteatomas.

Quaranta et al.^15^ compared the histomorphological characteristics of the perimatrix in surgical specimens removed from 30 patients aged less than 16 years and of 30 adults. The results showed that in children, the perimatrix is rich in mononuclear inflammatory cells with evident activity of collagenase enzyme, but in adults they were less abundant. Based on these data, the authors proposed that the characteristics of the perimatrix may play an important role in the pathogenesis of the cholesteatoma, suggesting that these histomorphological characteristics would influence the recurrence and invasion of pediatric cholesteatomas.

The only treatment known for cholesteatoma is a surgery named mastoidectomy. There are basically two techniques Wall-Down and Wall-Up; both have advocators among the surgeons and there is no consensus about the best approach in children. Jansen^16^, Sheehy^2^ and Glasscock^5^ prefer the more conservative surgery (wall up) to preserve the anatomy and function of the middle ear structures. Jahnke and Falk^17^ and Palva^8^ are in favor of more aggressive management (wall down) to prevent occurrence of residual or recurrent disease and associated complications. The determination of more aggressiveness or not of pediatric cholesteatomas could add some subsidies for the choice of surgical approach to this age range.

Thus, the present study aimed at comparing the measurements of perimatrix thickness in acquired cholesteatomas in pediatric and adult patients.

## METHOD

It was a transversal comparative and contemporary study.

The patients included in the study were selected in the Ambulatory of Chronic Otitis Media, Hospital de Clínicas de Porto Alegre (AOMC-HCPA).

At AOMC-HCPA, in the first visit, the patient undergoes anamnesis, with a specific protocol in addition to otoscopy on both sides, collected in digital images through Video Digital Recorder.

**Figure 1:**
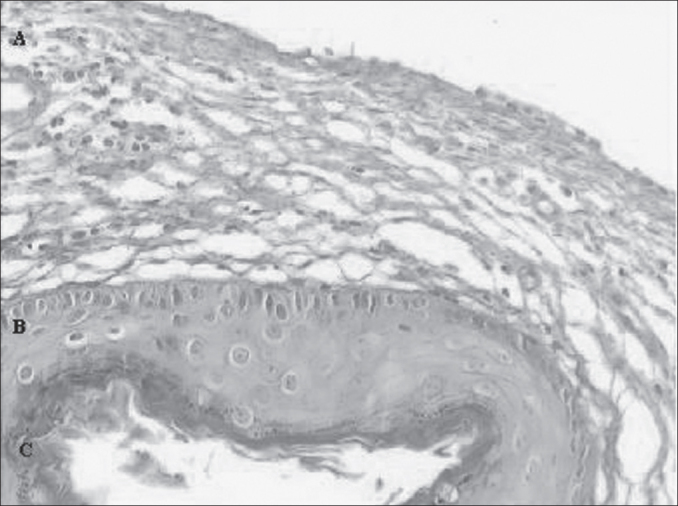
Digital images of the slide, with cross-section of the cholesteatoma, stained with Hematoxylin-Eosin. We can see three forming parts: A - A perimatrix - subepithelial connective tissue, containing collagen, elastic fibers, fibroblasts and inflammatory cells. B - A matrix - epithelium similar to normal skin epidermis. C - Cystic content - formed by keratin.

The inclusion of patients in this study followed the criteria below:

1. Diagnosis of cholesteatomatous chronic otitis media;
2. Histological presence of matrix and perimatrix in the material obtained from the collected cholesteatoma (Figure 1).

A classical case of histological representation of the matrix and perimatrix of the processed material can be seen in Figure 1. In A we can see the perimatrix comprised by subepithelial connective tissue, containing collagen and elastic fibers, fibroblasts and inflammatory cells. The matrix is identified by letter B, a similar epithelium to the epidermis of the normal skin. The cystic content, formed by keratin, is visualized in the region identified as C.

The exclusion criterion was:

1. Congenital cholesteatoma

The present study was approved by the Research and Post-Graduation group of HCPA in 2002. All patients that accepted to participate in the study signed the Free Informed Consent Term so that information could be anonymously used in scientific publications and for documentation and filing of images. Acceptance to sign the informed consent did not influence the treatment patients received.

We studied 74 cases of cholesteatoma collected in otological surgeries performed between May 2003 and July 2004, comprising 35 pediatric patients (0 to 18 years) and 39 adult patients (older than 18 years).

The material was collected by the otological surgeon, immediately fixed with formol at 10% and processed by habitual histological techniques, with inclusion in paraffin.

**Figure 2:**
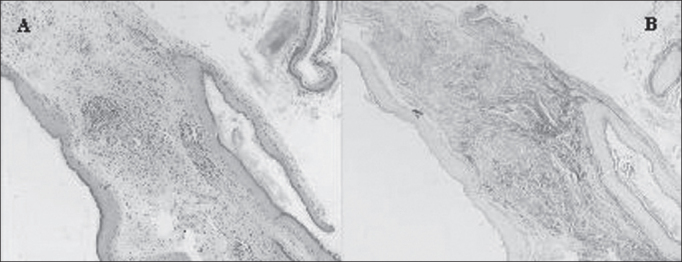
In this image we can see the same sample stained with HE (A) and Picrosirius (B).

We prepared two slides for the morphological analysis of each sample. Slides were stained with Hematoxylin-Eosin (HE) and Picrosirius (Sirius Red) and analyzed under optical microscope (Figure 2). We can notice that the contrast obtained with the second staining was much greater, because collagen fibers are stained in violet, which facilitates the differentiation of thickness to be measured. From each sample, 20 measurements of perimatrix thickness were made, reaching mean, median, minimum and maximum size, sum and delta (maximum minus minimum), which were the parameters used to compare both groups.

The reading of the material was blind and controlled by the main investigator.

**Figure 3:**
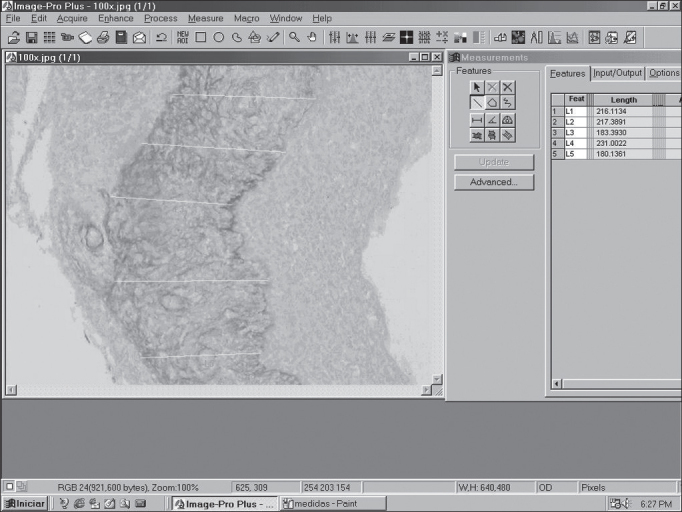
Image of the screen of software ImagePro Plus Media Cybernetics, showing a cross-section of cholesteatoma with the respective perimatrix measurements.

Perimatrix thickness was obtained through the analysis of computer images using the software ImagePro Plus Media Cybernetics (Figure 3). They comprised the results in pixel, which are corrected according to the increase used in the reading of the slides in the microscope to obtain the measurement in micrometers.

Spearman coefficient was used to check the correlation between the summed variables of perimatrix thickness and the age of the patient at the date of the surgery with the software SPSS 10.0 for Windows. We considered as statistically significant p values below 0.05.

## RESULTS

### Epidemiological data

The sample comprised 74 cholesteatomas collected from 69 patients, but 17 were excluded from the analysis of cholesteatoma.

In the group of patients excluded from the study, there were 7 who were aged up to 18 years, with mean ± standard deviation equal to 12.92 ± 3.77 years. Over the age of 18 years, there were 10 patients with mean age ± standard deviation equal to 34.41 ± 13.37 years. As to gender, there were 63% of male patients, 60% in the pediatric group and 67% in the adult groups.

Out of 52 included patients, the mean ± standard deviation of age was 24.08 ± 14.68. In this group, there were 25 patients aged up to 18 years, with mean ± standard deviation of 12.85 ± 3.63 years. Over 18, there were 27 patients whose mean age ± standard deviation was 33.69 ± 13.10 years. As to gender, the sample presented 51% of male patients. In the group of children this percentage went down to 59%, whereas in the adult group it was 42%.

### Perimatrix thickness

The mean values found are shown in Table 1, all expressed in micrometers. All medians are followed by interquartile interval (percentile 25 to percentile 75).

**Table 1:** Summed up variables of perimatrix thickness stratified by age range.

Parameter	Pediatric Group	Adult Group
	med (IQ) n = 28	med (IQ) n = 29
Mean	79 (41 a 259)	83 (26 a 174)
Median	77 (40 a 265)	68 (30 a 181)
Minimum	53 (16 a 165)	27 (12 a 100)
Maximum	1277 (64 a 398)	136 (53 a 280)
Delta	82 (44 a 248)	92 (45 – 190)
Sum	1.588 (831 a 5.185)	1.801 (558 a 3.867)

med (IQ) = Median and interquartile interval (percentile 25 to percentile 75), measurement in micrometers (mm)

**Figure 4:**
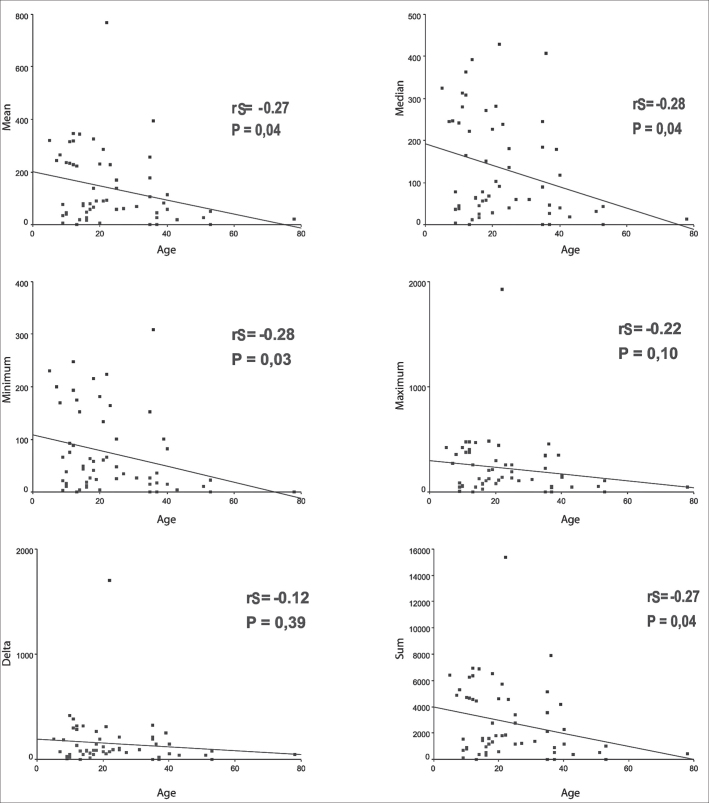
Graphs of Linear Regression between the ages and variables mean, median, minimum and maximum values, delta and sum.

Upon applying Spearman coefficient with age and variables: mean thickness, median, minimum and maximum value, delta and sum, we found poor to moderate correlations (Figure 4).

## DISCUSSION

Lim and Saunders^11^ were the first to present a detailed histological description of cholesteatomas. In this study they observed that cholesteatoma has keratinized stratified squamous epithelium, with four identical layers to the epidermis - basal, spinosum, granulosum and korneal - in addition to Langerhans cells in greater quantity than in the normal epidermal tissue and kerato-hyaline granules. This epithelium was named cholesteatoma matrix. They also observed the presence of connective loose tissue that contains collagen fibers, fibrocytes and inflammatory cells, which was named perimatrix; in most cases, it was in contact with the squamous or cylindrical ciliated cells, remaining of the middle ear mucosa. In some cases, despite the fact that the perimatrix was absent at optical microscopy, it was always present when studied under transmission electron microscopy, providing to be very thin, with collagen fibers that were practically absent and containing calcium carbonate crystals.

In the present study, we could confirm these findings. We found wide variability in thickness of the cholesteatoma perimatrix, both intra and inter-patients, in both groups. We also found five cases, four adult and one pediatric patients, in whom the perimatrix was extremely thin under optical microscopy (OM), reason why it was not possible to measure them. In these samples, thickness was considered equal to zero.

Pereira et al.^18^ compared cholesteatomas in patients that belonged to both age groups (20 adults and 11 children) to quantify the expression of cytokeratine^16^, which is a protein filament characteristic of hyperproliferative epithelia, but they did not detect differences between the age ranges. They found, as a casual finding, significantly greater number of cholesteatomas in adults that presented perimatrix visible under optical microscopy, when compared to children. In an attempt to explain this finding, they came up with the following hypotheses: the keratin content in pediatric cholesteatomas, because it has more pressure on the bone framework, would lead to reduction of the perimatrix, or, the reduction of the perimatrix could be a consequence of greater amount of collagenase, in pediatric cholesteatomas.

Taking into account that the action of collagenase could be one of the factors involved in bone erosion frequently found in cholesteatomatous otitis media and that there is clinical evidence to consider this pathology as more aggressive in children than in adults, we tried to determine, through objective measures, the thickness of the perimatrix in these two age groups and the reduction could be explained by smaller amounts of collagen fibers, and the reduction would, in turn, be resultant from collagenase action. However, differently from Pereira et al.^18^, we found inversed correlation between summed up measurements of perimatrix thickness and age.

The inversed correlation between perimatrix thickness and age redirects our discussion to other evidence. We cannot forget that the perimatrix is formed by many different elements, in addition to collagen fibers, and it is in this region of the cholesteatoma that we find the inflammatory elements.

Quaranta et al.^15^, based on clinical evidences, believe that pediatric cholesteatoma is more aggressive and could have less favorable prognosis. After this study, they suggested that the differences presented by age ranges could be related with histomorphometric characteristics of perimatrix and that it would play an important role in the pathogenesis of cholesteatoma. Confirming this hypothesis, Jacob et al.^19^ suggested that the growth of cholesteatoma is not stimulated by the matrix, but rather by the result of the inflammatory process, and that the perimatrix is the main pathogenic factor of cholesteatomas.

Milewski^20^ stated that the persistence of inflammation causes permanent scarring of the perimatrix, as well as the proliferation of fibroblasts and matrix, suggesting that cytokines (inflammation molecules and immune response) released by inflammatory cells, among other factors, could be responsible for the growth of cholesteatomas and for the bone destruction caused by it. Marenda and Aufdermorte^21^ reported the immune-localization of five different cytokines in the perimatrix.

In our analysis we found indications, through statistically significant Spearman correlation coefficient that the thickness of the perimatrix reduces with aging of patients. As previously mentioned, it is in the perimatrix that we have the inflammatory process in which we can cause formation of granulation tissue that, in turn, activates bone reabsorption through increase in activity of osteoclasts. Ossicle erosion is frequently associated with presence of chronic otitis media cholesteatoma^14^^,^^22^^,^^23^. Based on Lazarus et al.^24^ findings that stated that the chemicalactivity of the perimatrix had a role in bone absorption, Gantz et al.^25^ demonstrated that inflammatory mononuclear cells produce collagenolytic enzymes. The importance of the perimatrix in bone reabsorption is also pointed out by Abramson et al.^26^ and it is the first to suggest that the potential of infiltration of cholesteatoma would be related with chemoenzymatic activity of perimatrix.

We can make an analogy between perimatrix and a “battlefield” in which there would be competition for middle ear territory. On the one side, to attack, we have the cholesteatoma; on the other side, adjacent tissues of tympanic cavity mucosa. As there is expansion of cholesteatoma, inflammatory reaction increases, consequently producing more elements of the inflammatory cascade. In our opinion, it is in the perimatrix and in the processes that occur in it that lies the aggressiveness of the cholesteatoma. Thus, the different clinical characteristics of pediatric cholesteatomas would be related with exuberance of its inflammation.

Despite the fact that the technique used in the present study was precise to measure perimatrix thickness, it is not specific to detect which elements are reduced in the structure. To that end, the authors intend to follow on with studies to further quantify the elements that are part of the perimatrix of acquired cholesteatomas.

## CONCLUSION

There is evidence in this sample that there is mild to moderate inversed correlation between thickness of perimatrix of acquired cholesteatoma and patients’ age at the surgery date.
